# Anti-tuberculosis chemotherapy alters TNFR2 expression on CD4+ lymphocytes in both drug-sensitive and -resistant tuberculosis: however, only drug-resistant tuberculosis maintains a pro-inflammatory profile after a long time

**DOI:** 10.1186/s10020-021-00320-4

**Published:** 2021-07-14

**Authors:** Norma A. Téllez-Navarrete, Lucero A. Ramon-Luing, Marcela Muñoz-Torrico, Mario Preciado-García, Karen Medina-Quero, Rogelio Hernandez-Pando, Leslie Chavez-Galan

**Affiliations:** 1 Laboratory of Integrative Immunology, Instituto Nacional de Enfermedades Respiratorias “Ismael Cosío Villegas”, Calzada de Tlalpan No. 4510, CP. 14080 Mexico City, Mexico; 2 Clinic of Tuberculosis, Instituto Nacional de Enfermedades Respiratorias “Ismael Cosío Villegas”, Mexico City, Mexico; 3 Laboratory of Immunology, Escuela Militar de Graduados en Sanidad, Mexico City, Mexico; 4 Experimental Pathology Section, Department of Pathology, Instituto Nacional de Ciencias Médicas y Nutrición Salvador Zubirán, Mexico City, Mexico

**Keywords:** Tuberculosis, Drug-resistance, Tregs, Receptors, TNF

## Abstract

**Background:**

Tuberculosis (TB) is an infectious disease. During TB, regulatory T cells (Treg) are related to poor prognosis. However, information about conventional and unconventional Treg (cTreg and uTreg, respectively) is limited. The tumour necrosis factor (TNF) and its receptors (TNFR1 and TNFR2) are necessary for mycobacterial infection, and TNFR2 signalling is required to maintain Treg.

**Methods:**

A blood sample of drug-susceptible (DS-TB) and drug-resistant tuberculosis (DR-TB) patients was obtained before (basal) and after 2 and 6 months of anti-TB therapy. Expression of TNF, TNFR1, and TNFR2 (transmembrane form, tm) on cTreg, uTreg, activated CD4+ (actCD4+), and CD4+ CD25− (CD4+) T cell subpopulations were evaluated. The main objective was to identify immunological changes associated with sensitive/resistant Mtb strains and with the use of anti-TB therapy.

**Results:**

We found that after 6 months of anti-TB therapy, both DS- and DR-TB patients have decreased the frequency of cTreg tmTNF+, CD4+ tmTNFR1+ and CD4+ tmTNFR2+. Nevertheless, after 6 months of therapy, only DR-TB patients decreased the frequency of actCD4+ tmTNF+ and actCD4+ tmTNFR2+, exhibited a systemic inflammatory status (high levels of TNF, IFN-γ and IL-12), and their purified CD4+ T cells showed that TNF and TNFR2 are up-regulated at the transcriptional level. Moreover, DS- and DR-TB down-regulated TNFR1 and other proteins associated with Treg (FOXP3 and TGFβ1) in response to the anti-TB therapy.

**Conclusion:**

These results partially explain the differences in the immune response of DS-TB vs DR-TB. The frequency of actCD4+ tmTNFR2+ cells and inflammatory status should be considered in the follow-up of therapy in DR-TB patients.

**Supplementary Information:**

The online version contains supplementary material available at 10.1186/s10020-021-00320-4.

## Background

Tuberculosis (TB) is an infectious disease caused by the bacilli *Mycobacterium tuberculosis* (Mtb). The last statement of the World Health Organization (WHO) reported 10 million TB cases worldwide. Unfortunately, drug-resistant tuberculosis (DR-TB) cases have increased; WHO estimated that in 2018, there were about half a million persons worldwide who developed TB resistance to rifampicin and rifampicin/isoniazid (World Health Organization 2019). DR-TB requires a long therapy period, and the drugs used have higher toxicity than those used in drug-sensitive tuberculosis (DS-TB).

Regulatory T cells (Treg) belong to a specialised subpopulation that suppresses immune response through the delivery of cytokines like interleukin (IL)-10, IL-35 and transforming growth factor-beta (TGFβ). Classically, Treg expresses the transcription factor forkhead box P3 (FOXP3) (Huehn and Beyer 2015; Sabbagh et al. 2018). Treg cells are divided into two subpopulations: (1) “conventional” or natural Treg cells (cTreg), which are phenotypically CD4+ CD25+ FOXP3+ and, (2) “unconventional” Treg cells (uTreg), which are negative for CD25 expression (CD4+ CD25-FOXP3+); albeit both Treg subpopulations have the inhibitory capacity to suppress cell proliferation. The origin of the uTreg subpopulation is controversial, and some authors propose that they are immature Treg cells, and others suggest that uTreg cells are effector CD4+ T cells with a transient expression of FOXP3 (Tiwari 2018; Yang et al. 2009; Angerami et al. 2017).

DR-TB increases the frequency of Treg and is associated with the development of cavitary pulmonary lesions. Reports showed that the resection of pulmonary cavities is related to a decrease in the frequency of Treg (Churina et al. 2012; Wu et al. 2010). Likewise, it has been reported that plasma levels of IL-10 and TGFβ are higher in DR-TB than in DS-TB patients, and recent reports have shown that extensively Drug-resistant tuberculosis (XDR-TB) patients with failed anti-TB therapy had increased the frequency of Treg compared to DS-TB (Li et al. 2015; Davids et al. 2018). This data suggests that the Treg subpopulation plays an essential role in inducing an immune dysfunction during TB.

Tumour necrosis factor (TNF) is a pro-inflammatory cytokine necessary to activate and maintain granuloma formation during TB (Dorhoi and Kaufmann 2014). TNF is synthesised as a precursor in a transmembrane form (tmTNF), and under any activation stimuli, tmTNF is cleaved by the TNF-α converting enzyme (TACE) to induce TNF soluble form (solTNF). Both tmTNF and solTNF interact with TNF receptor 1 or 2 (TNFR1 and TNFR2, respectively). Moreover, TNFR1 and TNFR2 can also be found in either soluble or transmembrane form (Garcia 2011)_._

The pro-inflammatory function of the TNF pathway is clear. However, this pathway also has been related to immune regulatory functions. For instance, evidence shows that the TNF pathway is necessary to activate and expand Treg cells through TNFR2 (He et al. 2018; Okubo et al. 2013; Lubrano di Ricco et al. 2020). Within the TB context, reports suggest that TNFRs control exacerbated inflammation, whereas TNFR1 is necessary to recruit immune cells at the mycobacterial infection site, and tmTNFR2 expression is necessary to induce the regulatory function in myeloid-derived suppressor cells (Chavez-Galan et al. 2016, 2017, 2019; Uysal et al. 2018).

To date, the expression of TNF pathway molecules has not been described on the cell surface of CD4+ T cell subpopulations from TB patients or how the expression of these molecules could influence the frequency and function of Treg. This study's main goal was to determine the expression profile of TNF-pathway molecules on CD4+ T cells from TB patients and to confirm if this may influence the frequency of Treg. Blood samples from DR- and DS-TB patients were obtained before receiving the anti-TB therapy and then after 2 and 6 months of therapy to confirm if Mtb strains (DR and DS) or anti-TB treatment induced an altered expression of the TNF pathway molecules.

Our results showed that TNF pathway molecules expressed on the CD4+ T cell subpopulation contribute to immune dysfunction in DR-TB patients. We observed that after 6 months of anti-TB therapy, both DS- and DR-TB patients, the frequency of cTreg tmTNF+, CD4+ tmTNFR1+ and CD4+ tmTNFR2+ cells decreases. However, even with 6 months of anti-TB therapy, only DR-TB decreases the frequency of actCD4+ tmTNF+ and actCD4+ tmTNFR2+ cells, exhibiting a systemic inflammatory status characterised by high levels of TNF, IFN-γ, and IL-12. This inflammatory status is probably a consequence of up-regulation at the transcriptional level of TNF, and TNFR2 observed in total CD4+ T cells from these patients. Our results provide new highlights in searching for better molecules to follow-up DR-TB patients, and it is one of the core activities prioritised by the WHO to eliminate TB.

## Materials and methods

### Ethical approval

All procedures performed in the study were conducted following the principles stipulated in the Helsinki Declaration.

This study was approved by the Ethics committee of the Instituto Nacional de Enfermedades Respiratorias “Ismael Cosio Villegas” (protocol code B07-18). All patients provided written informed consent to use their blood samples and clinical data for research. Sample from healthy donors was obtained from the institutional blood bank (leukocytes fraction).

### Patients and blood samples

The study population consisted of twenty patients with active pulmonary TB; who were enrolled during 2018 at the Instituto Nacional de Enfermedades Respiratorias Ismael Cosio Villegas, Mexico City, Mexico. Patients with comorbidities, such as HIV infection, malignant diseases, chronic renal failure, and liver cirrhosis, were excluded.

Patients were classified into two groups: thirteen were diagnosed as DS-TB patients, and seven were DR-TB patients. The diagnosis was made according to PCR Xpert-MTB/RIF probes' results to detect resistance to rifampicin. Drug-sensitivity testing to first and second-line anti-TB drugs was evaluated using the BD BACTEC™ MGIT™ systems recommended by the manufacturer, and the results of these assays were confirmed using an LJ medium.

Patients received anti-TB therapy following the international normative and had a clinical follow-up. Blood samples used for experiments developed in this study were collected when a diagnosis was done (basal sample) and after 2 and 6 months of anti-TB therapy (2 m and 6 m, respectively). These times correspond in DS-TB, to the end of the intensive phase and the end of the maintenance phase of the treatment (2 m and 6 m, respectively); whereas in DR-TB, these times corresponded to the intensive phase and the beginning of the maintenance phase of the treatment (2 m and 6 m, respectively). Furthermore, it is essential to mention that 12 (92%) DS-TB and 6 (86%) of DR-TB patients had an acid-fast bacillus (AFB) smear-negative test in the second month.

Finally, data of clinical parameters evaluated in peripheral blood were obtained from Electronic Medical Records (EMR), several laboratory tests were performed as part of the integral clinical follow-up that was done to each patient.

### Peripheral blood mononuclear cells (PBMCs)

Blood samples of patients were obtained using BD Vacutainer CPT™ (BD Biosciences, San Jose, CA, USA) tubes before starting treatment (basal sample) and then at 2 m and 6 m after anti-TB therapy. PBMCs were isolated within one hour of the blood draw, immediately cryopreserved and subsequently used for multiparametric cytometric analysis and isolation of CD4+ T cells. Plasma was obtained and stored at − 70° until use. PBMCs of healthy donors were isolated from buffy coats by standard LymphoprepTM (Accurate Chemical-Scientific, Westbury, NY, USA) gradient centrifugation. Posteriorly, these PBMCs were used to isolate CD4+ T cells, as is indicated below.

### Multiparametric cytometry analysis

A single-cell suspension of PBMCs was prepared to determine the cellular phenotype by flow cytometry. The following monoclonal antibodies (mAb) were used: CD3 (clone HIT3a), CD4 (clone A161A1), CD25 (clone BC96), TNF (clone MAb11) TNFR1 (clone W15099A), TNFR2 (clone 3G7A02), CD45RA (clone HI100), and CCR7 (clone G043H7), All the mAbs were provided by BioLegend (San Diego, CA, USA). PBMCs were stained for 30 min at 4 °C in the dark.

To determine the intracellular expression of FOXP3, PBMCs were washed, fixed, and permeabilised with the Kit True Nuclear™ Transcription Factor Buffer Set (BioLegend). Then cells were washed twice and stained with mAb to identify FOXP3 (clone 206D). Finally, cells were washed and resuspended in 1% paraformaldehyde.

Cells used for Fluorescence Minus One (FMO) condition were stained and acquired in parallel to identify staining background levels, and dead cells were omitted using Zombie Red™ (BioLegend) viability kit.

The data were collected using a FACS Aria II (BD Biosciences, San Jose, CA, USA) and then analysed by FlowJo v10.2 (FlowJo LLC, Inc, Ashland, OR, USA). In each case, 50,000 events of the live cell's gate were acquired per sample.

### Cytokine quantification

Soluble level of TNF, IFNγ, IL-10 and IL-12 p70 (provided by BioLegend), TGFβ1 (R&D Systems) and IL-35 (MyBioSource) were measured in plasma by Enzyme-Linked Immunosorbent Assay (ELISA). All the proteins were quantified following the manufacturer's instructions. Absorbance values were obtained using the iMark™ (Microplate Absorbance Reader from Bio-Rad) and software Microplate Manager V6.

### Isolation of CD4+ T cells

According to the manufacturer's instructions, CD4+ T cells were isolated by negative selection using the Human CD4+ T cell isolation kit (Miltenyi Biotec, Germany). Enrichment of CD4+ T cells fraction was routinely > 96%, as analysed by flow cytometry.

### RNA extraction and reverse transcription

For qPCR assays, RNA extraction and the cDNA synthesis were performed, as previously reported (Rodriguez-Cruz et al. 2019). Briefly, total RNA from purified CD4+ and CD4− T cells from TB patients and control groups were obtained with the RNeasy Mini Kit (Qiagen, Hilden, Germany) following the manufacturer’s instructions. Genomic DNA contamination was removed with RNA-Free DNAse Set (Qiagen). The Amount of RNA was evaluated by Qubit™ assay kit in the Qubit 2.0 Fluorometer (Life Technologies, Waltham, USA). For cDNA synthesis, 60 ng of total RNA were converted with the High Capacity cDNA Reverse Transcription Kit (Applied Biosystems, Waltham, USA) in a volume of 20 μl according to the manufacturer's guidelines.

### Quantitative polymerase chain reaction

Quantitative real-time PCR was performed as previously we reported (Rodriguez-Cruz et al. 2019), using TaqMan probes specific for the target genes: TNF (Hs00174128_m1), TNFR1 (Hs01042313_m1), TNFR2 (Hs00961750_m1), FOXP3 (Hs01085834_m1), TGFβ1 (Hs00248373_m1), and EBI3 (Hs01057148_m1). ACTB (β-actin) (Hs01060665_g1) and 18S (18S ribosomal RNA gene) (Hs03928990_g1) were used as endogenous controls. Singleplex reactions were prepared with the Maxima Probe/ROX qPCR Master Mix (Thermo Fisher Scientific, Waltham, USA), and all amplifications were run by duplicate under the following thermal conditions: 95 °C for 10 min followed by 40 cycles of 60 °C for 1 min and 95 °C for 15 s, with the StepOnePlus™ Real-Time PCR Systems (Applied Biosystems). The relative expression of transcripts was quantified using the ΔΔCT method. To obtain the results first was calculated the n-fold change for each target gene in each experimental condition, normalised with the endogenous controls ACTB and 18S, and relative to the control group. CD4+ T cells from 3 healthy donors were included as the control group (2^−ΔΔCT^ = 1) and used as a reference; this means the relative expression of DR- and DS-TB at the basal time was calculated taking the reference of gene expression of the healthy donors. Then, we calculated the fold change percentage by obtaining the difference between 6 months and before of anti-TB therapy (6 m and 0 m, respectively) per each group (DR- and DS-TB). The gene expression in each patient group at 6 m is relative to their 0 m time.

### Statistical analysis

Data are shown as mean with standard deviation (SD) or median with interval interquartile (IQR, 25–75). The data normality was tested using the D'Agostino test. The one-sided Fisher's exact test was used in dichotomous variables. The difference between the two groups was tested using U Mann–Whitney or Wilcoxon matched-pairs test. One-Way ANOVA test with Dunnet’s multiple comparisons post hoc analysis was used to compare more than two groups, and *p* values are two-tailed and unadjusted for multiple comparisons. Statistical analysis was performed using GraphPad Prism V 8.4.3 (GraphPad Software, La Jolla, CA).

## Results

### Baseline characteristics of the study population

Twenty patients with pulmonary TB diagnosis were enrolled in this study; thirteen patients were diagnosed as DS-TB and seven as DR-TB individuals (Table 1). The drug profile of DR-TB patients was: 58% mono-resistant to rifampicin, and 42% were resistant to at least one extra drug (Additional file 1: Table S1); therefore, in this study, the DR-TB group includes patients resistant to one or more drugs.

**Table 1:** Demographic and clinical characteristics of the study population before the use of anti-TB therapy

	DS-TB (N = 13)	DR-TB (N = 7)	p
Age (years)	33 (25–44)	56 (27–67)	0.057
Male, n(%)	6 (46%)	4 (57%)	0.500
Diabetes, n(%)	5 (48%)	4 (57%)	0.370
Glucose, mg/dL	106 (94–151)	132 (93–146)	0.641
Sputum culture			
* Mycobacterium tuberculosis*	12 (92%)	6 (86%)	–
* Mycobacterium bovis*	1 (8%)	1 (14%)	–

Data is represented with median and interquartile range (IQR, 25–75). U Mann Whitney and one-sided Fisher’s exact test were used

*DS-TB* drug-susceptible tuberculosis, *DR-TB* drug-resistant tuberculosis

DR- and DS-TB patients groups had a similar distribution of age and gender. Moreover, both groups had diabetes mellitus as the most frequent comorbidity and showed similar glucose levels (Table 1). At the time of diagnosis (basal sample): 12 (92%) DS-TB and 6 (86%) DR-TB patients had a positive Mtb culture. Although one (8%) DS-TB and one (14%) DR-TB patients had a positive *M. bovis* culture (Table 1), they did not display different results of the proteins evaluated in this study compared to the patients with an Mtb positive culture.

Absolute cell count was a clinical parameter evaluated as well. Data showed that total leukocytes and the individual account of neutrophils, lymphocytes, and monocytes decreased at 2 m compared to basal. However, the number of cells at 6 m was similar to 2 m, which means that the significant change in the total cell count of peripheral blood is observed at 2 m, which is also when almost all patients had an AFB smear-negative test (Table 2).

**Table 2:** Absolute cells count in peripheral blood at basal, 2 m and 6 m of anti-TB therapy

Absolute cells count	DS-TB (N = 13)	DR-TB (N = 7)
Basal		
Leukocytes, media (SD), (RV 4–10 × 10^3^ cells/mm^3^)	11.5 (3.40)	9.98 (3.03)
Neutrophils, media (SD), (RV 2–7.5 × 10^3^ cells/mm^3^)	8.95 (3.5)	7.67 (2.87)
Lymphocytes, media (SD), (RV 1–4 × 103 cells/mm^3^)	1.72 (0.78)	1.6 (0.33)
Monocytes, media (SD), (RV 0.2–1 × 10^3^ cells/mm^3^)	0.87 (0.48)	0.66 (0.24)
2 months of anti-TB therapy		
Leukocytes, media (SD), (RV 4–10 × 10^3^ cells/mm^3^)	6.75 (2.39)	6.8 (1.85)
Neutrophils, media (SD), (RV 2–7.5 × 10^3^ cells/mm^3^)	4.0 (2.36)	4.47 (1.41)
Lymphocytes, media (SD), (RV 1–4 × 10^3^ cells/mm^3^)	1.80 (0.86)	1.45 (0.57)
Monocytes, media (SD), (RV 0.2–1 × 10^3^ cells/mm^3^)	0.51 (0.31)	0.72 (0.19)
6 months of anti-TB therapy		
Leukocytes, media (SD), (RV 4–10 × 10^3^ cells/mm^3^)	6.8 (1.86)	6.35 (1.50)
Neutrophils, media (SD), (RV 2–7.5 × 10^3^ cells/mm^3^)	4.03 (1.53)	4.07 (1.16)
Lymphocytes, media (SD), (RV 1–4 × 10^3^ cells/mm^3^)	2.01 (0.69)	1.41 (0.41)
Monocytes, media (SD), (RV 0.2–1 × 10^3^ cells/mm^3^)	0.56 (0.24)	0.62 (0.13)

*DS-TB* drug-susceptible tuberculosis, *DR-TB* drug-resistant tuberculosis, *SD* standard deviation, *RV* reference value (provided by the institutional Clinical Laboratory)

### DS-TB and DR-TB decrease the frequency of cTreg and cTreg tmTNF+ cells after 6 months of anti-TB therapy

CD4+ T cells are divided into several subpopulations in agreement with different markers expressed on the cell surface. Peripheral blood from TB patients underwent flow cytometry; once the live cells were identified, the CD3+ CD4+ cell gate was limited (Additional file 1: Fig. S1A). Subsequently, the presence of CD25 and FOXP3 were measured, and four subpopulations of CD4+ T cells were identified: (1) activated CD4+ T cells (CD3+ CD4+ CD25+ FOXP3−, hereafter called actCD4+), (2) cTreg cells (CD3+ CD4+ CD25+ FOXP3+), (3) uTreg cells (CD3+ CD4+ CD25-FOXP3+), and (4) other CD4+ T cells (CD3+ CD4+ CD25− FOXP3−, hereafter called CD4+ T cells) (Q1, Q2, Q3 and Q4 respectively, Additional file 1: Fig. S1A). Finally, expression of tmTNF, tmTNFR1 and tmTNFR2 was evaluated in each CD4+ T cell subpopulation (Additional file 1: Fig. S1B).

**Fig. 1:**
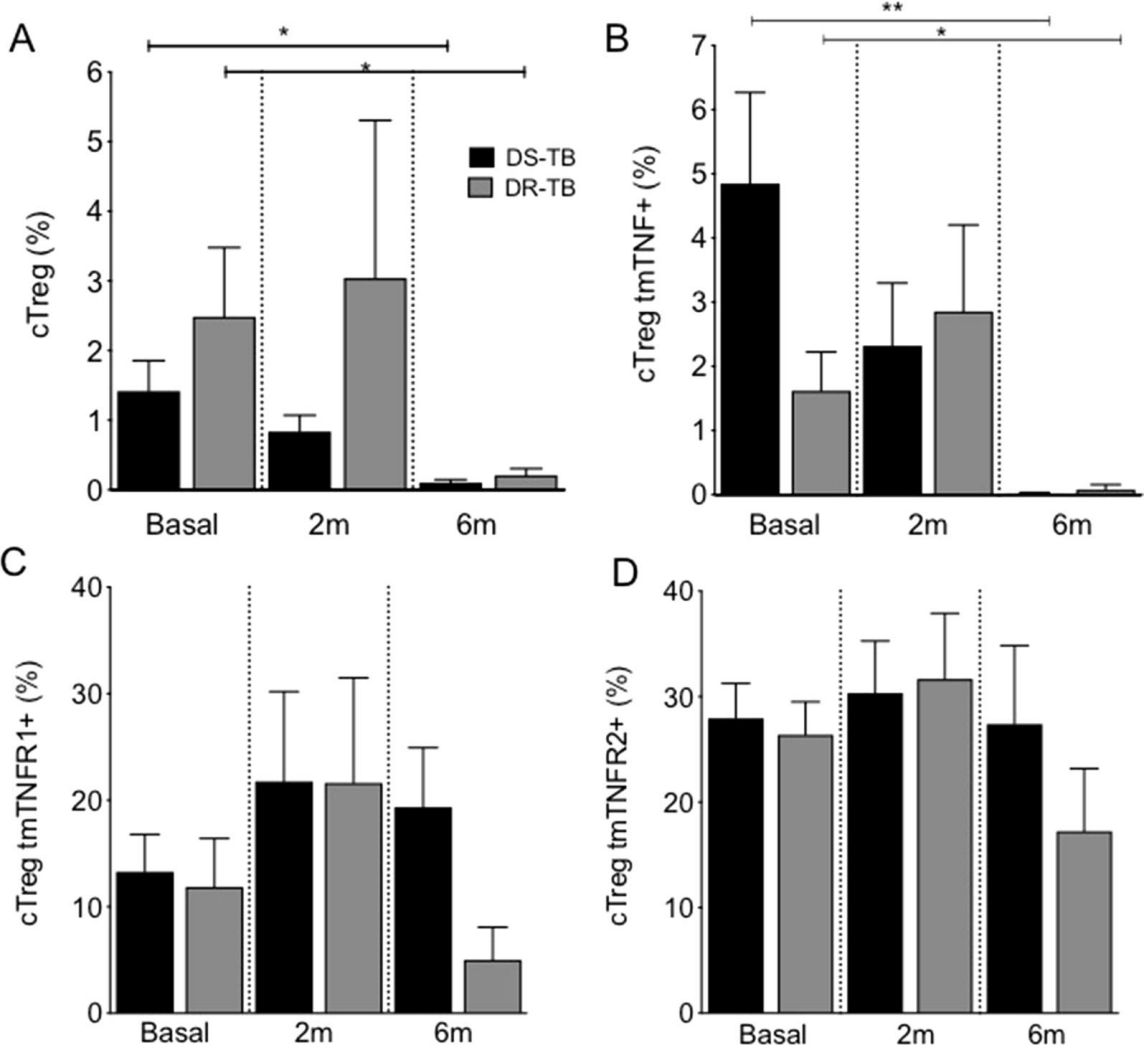
cTreg and cTregTNFR + are decreased in DS-TB and DR-TB patients in response to anti-TB therapy. Peripheral mononuclear cells from drug-susceptible (DS-TB) and drug-resistance (DR-TB) tuberculosis patients were obtained at diagnosis time (basal), two months (2 m), and 6 months (6 m) of use of anti-TB therapy, and then they were prepared for flow cytometry. For analysis, a gate of CD3+ CD4+ CD25+ Foxp3+ cells (conventional regulatory T cells, cTreg) was identified. Then, the expression of tmTNF, tmTNFR1 and tmTNFR2 on the cTreg cell surface was evaluated. **A** The frequency of cTreg cells was obtained at basal, 2 m and 6 m of anti-TB therapy. The frequency of cTreg positive to tmTNF (**B**), tmTNFR1 (**C**) and tmTNFR2 (**D**) was evaluated. DS-TB n = 11, DR-TB n = 7. Bar graphs showing the mean ± SD. One Way ANOVA with Dunn's post-test multiple comparisons tests (*p < 0.05 **p < 0.01)

Our data showed that cTreg cells' frequency decreases significantly at 6 m in both DS-TB and DR-TB patients, compared to basal time (Fig. 1A). Expression of TNF pathway molecules was evaluated on cTreg cell surface, and the frequency of cTreg tmTNF+ also decreased at 6 m in both TB groups (Fig. 1B). However, neither tmTNFR1 and tmTNFR2 expression on cTreg cells was different at the basal time and after 6 m of anti-TB therapy (Fig. 1C and D). Our result suggests that anti-TB therapy decreases cTreg and cTreg tmTNF+ cells' frequency in both DS-TB and DR-TB patients.

### DS-TB decreases the frequency of uTreg tmTNFR2+cells after 2 months of anti-TB therapy

**Fig. 2:**
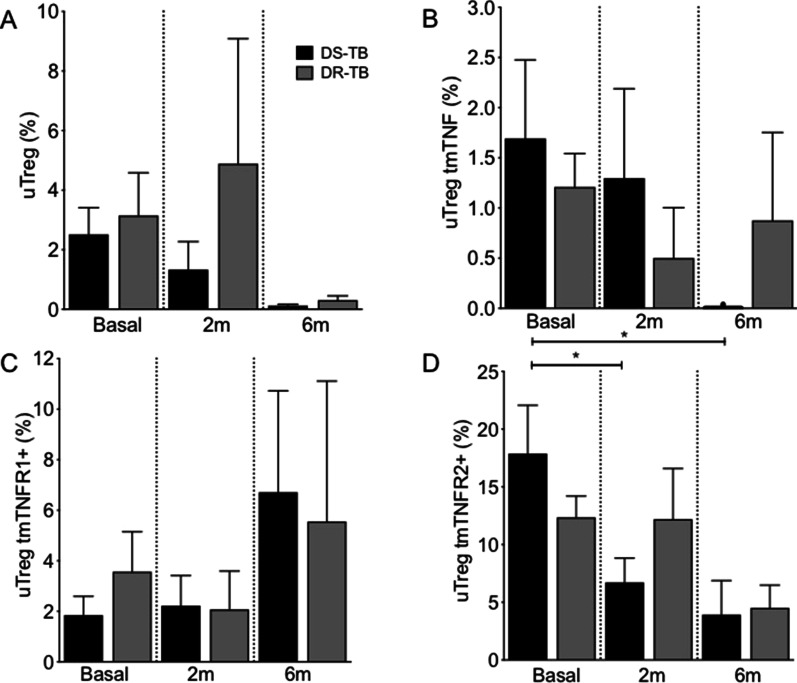
uTreg TNF+ and uTreg TNFR2+ are decreased in DS-TB patients in response to anti-TB therapy. Peripheral mononuclear cells from drug-susceptible (DS-TB) and drug-resistance (DR-TB) tuberculosis patients were obtained at diagnosis time (basal), 2 months (2 m), and 6 months (6 m) of anti-TB chemotherapy, and then they were prepared for flow cytometry. For analysis, a gate of CD3+ CD4+ CD25-Foxp3+ cells (unconventional regulatory T cells, uTreg) was identified. Then, the expression of tmTNF, tmTNFR1 and tmTNFR2 on the uTreg cell surface was evaluated. **A** The frequency of uTreg cells was obtained at basal, 2 m and 6 m of anti-TB therapy. The frequency of uTreg positive to tmTNF (**B**), tmTNFR1 (**C**) and tmTNFR2 (**D**) was evaluated. DS-TB n = 11, DR-TB n = 7. Bar graphs showing the mean ± SD. One Way ANOVA with Dunn's post-test multiple comparisons tests (*p < 0.05)

Anti-TB therapy did not affect the frequency of uTreg cells. Although we observed a decrease in uTreg cells' frequency at 6 m, this was not statistically significant (Fig. 2A). Similarly, the frequency of uTreg tmTNF+ and uTreg tmTNFR1+ cells was not affected. Even after 6 m, DS-TB patients displayed a tendency to decrease uTreg tmTNF+ cells and increase uTreg tmTNFR1+ cells, even though there were no statistically significant differences (Fig. 2B and C, respectively). Finally, after 2 m of anti-TB therapy, DS-TB decreased the frequency of uTreg tmTNFR2+ (Fig. 2D). Together, this data suggests that anti-TB therapy decreases the frequency of uTreg tmTNFR2+ cells only during DS-TB treatment.

### DR-TB decreases the frequency of actCD4+ tmTNF+ and actCD4+ tmTNFR2+ cells at the 6th month of anti-TB therapy

**Fig. 3:**
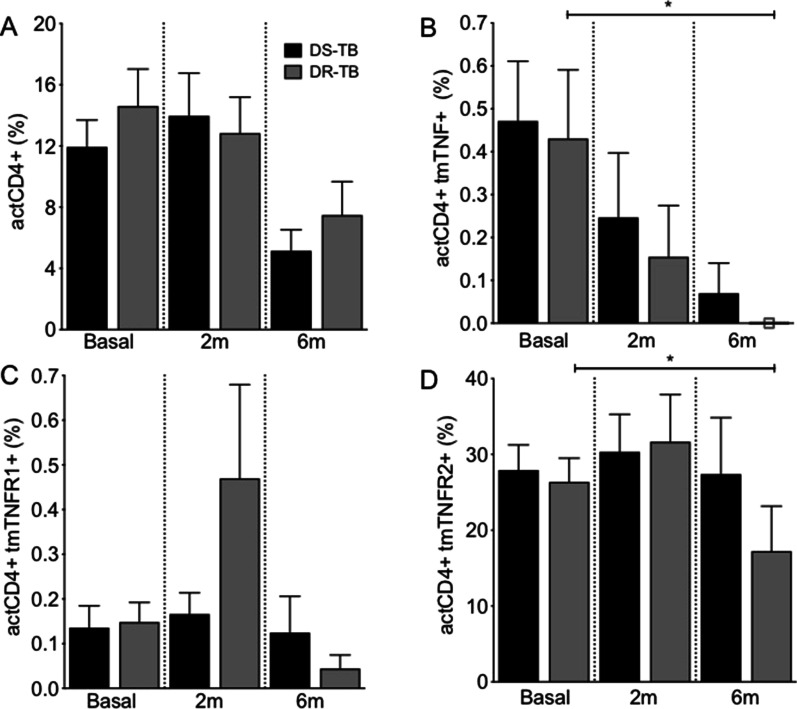
Frequency of actCD4+ tmTNF+ and actCD4+ TNFR2+ T cells decrease in response to anti-TB therapy in DR-TB. Peripheral mononuclear cells from drug-susceptible (DS-TB) and drug-resistance (DR-TB) tuberculosis patients were obtained at diagnosis time (basal), 2 months (2 m), and 6 months (6 m) of use of anti-TB therapy, and then they were prepared for flow cytometry. For analysis, a gate of CD3+ CD4+ CD25+ Foxp3− cells (activated CD4+ T cells, actCD4+) was identified. Then, the expression of tmTNF, tmTNFR1 and tmTNFR2 on actCD4+ cell surface was evaluated. **A** The frequency of actCD4+ cells was obtained at basal, 2 m and 6 m of anti-TB therapy. Frequency of actCD4+ cells positive to tmTNF (**B**), tmTNFR1 (**C**) and tmTNFR2 (**D**) were evaluated. DS-TB n = 11, DR-TB n = 7. Bar graphs showing the mean ± SD. One Way ANOVA with Dunn's post-test multiple comparisons tests (*p < 0.05)

The frequency of actCD4+ and actCD4+ tmTNFR1+ cells are not affected in response to anti-TB therapy (Fig. 3A and C, respectively), but the frequency of actCD4+ tmTNF+ and actCD4+ tmTNFR2+ cells decreased at 6 m of therapy during DR-TB treatment, but not during DS-TB treatment (Fig. 3B and D, respectively). Thus, our data show that anti-TB therapy decreases the frequency of actCD4+ tmTNF+ and actCD4+ tmTNFR2+ cells only during DR-TB treatment.

### DS-TB and DR-TB increase the frequency of CD4+ T cells but decrease CD4+ tmTNFR1+ and CD4+ tmTNFR2 cells in the 6th month of anti-TB therapy

**Fig. 4:**
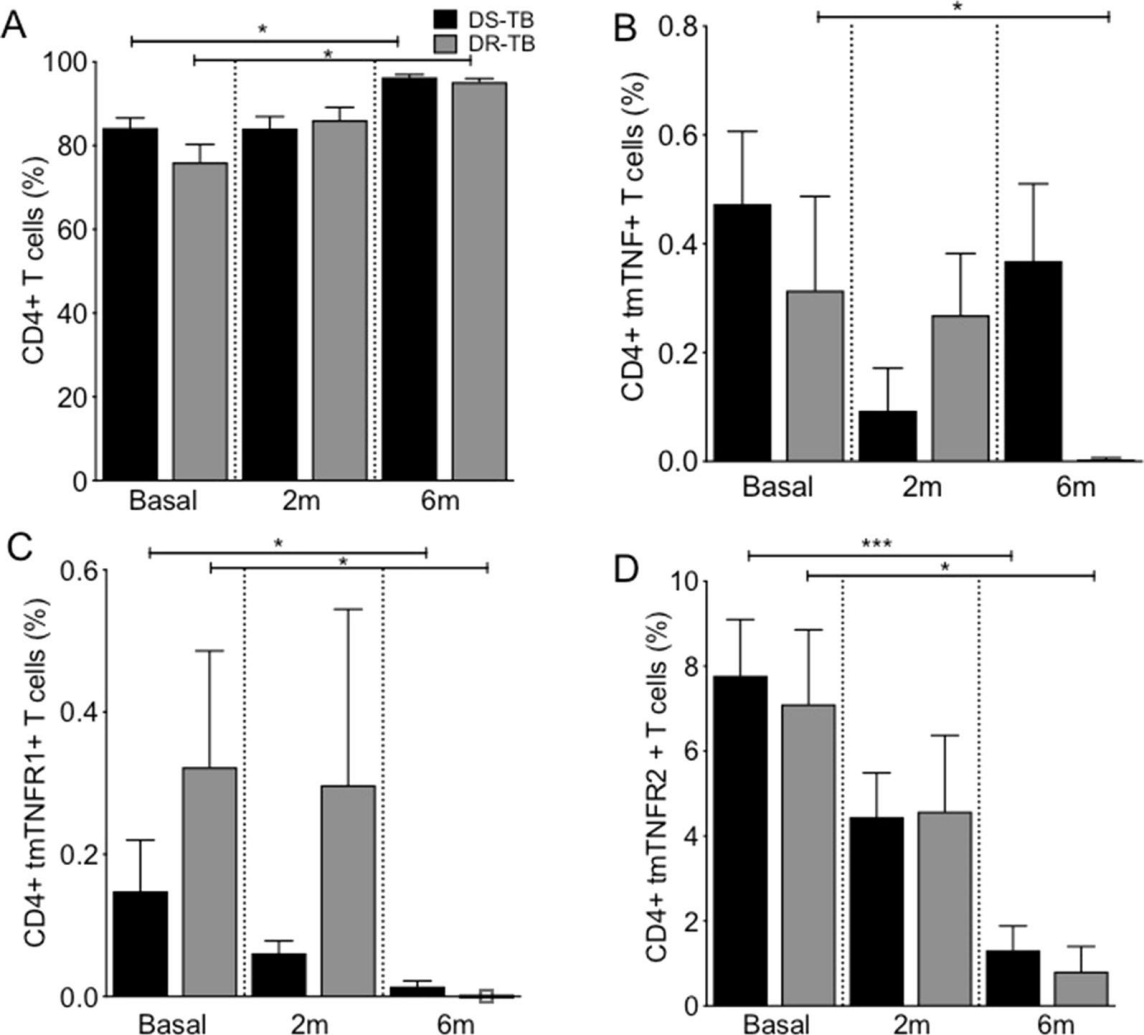
In response to anti-TB therapy, DR-TB increases CD4+ T cells but decreases the expression of TNF molecules pathway. Peripheral mononuclear cells from drug-susceptible (DS-TB) and drug-resistance (DR-TB) tuberculosis patients were obtained at diagnosis time (basal), 2 months (2 m), and 6 months (6 m) of use of anti-TB therapy, and then they were prepared for flow cytometry. For analysis, a gate of CD3+ CD4+ CD25-Foxp3− cells (CD4+ T cells) was identified. Then, the expression of tmTNF, tmTNFR1 and tmTNFR2 on CD4+ T cell surface was evaluated. **A** The frequency of CD4+ T cells was obtained at basal, 2 m and 6 m of anti-TB therapy. The frequency of CD4+ T cells positive to tmTNF (**B**), tmTNFR1 (**C**) and tmTNFR2 (**D**) was evaluated. DS-TB n = 11, DR-TB n = 7. Bar graphs show the mean ± SD. One Way ANOVA with Dunn's post-test multiple comparisons tests (*p < 0.05, ***p < 0.001)

Finally, the subpopulations of CD4+ T cells (Additional file 1: Fig. S1A) were evaluated. Results showed that both DS- and DR-TB patients increased the frequency of CD4+ T cells after 6 m of therapy (Fig. 4A). Although only DR-TB decreased the frequency of CD4+ tmTNF+ T cells at 6 m (Fig. 4B), both DS- and DR-TB decreased the frequency of CD4+ tmTNFR1+ T cells and CD4+ tmTNFR2+ T cells at 6 m (Fig. 4C and D, respectively).

We hypothesised that TB patients have a predominance of the effector cell subpopulation because we observed an increase in the frequency of CD4+ T cells as a response to anti-TB treatment, and the expression of the surface markers CD45 and C–C chemokine receptor 7 (CCR7) were evaluated by flow cytometry (Förster et al. 2008). First, we delimited the CD3+ CD4+ population; and then the expression of CD45RA and CCR7 were evaluated to identify: Naïve (CD45RA+/CCR7+), central memory (TCM, CD45RA−/CCR7+), effector memory (TEM, CD45RA−/CCR7−), and effector memory RA (TEMRA, CD45RA+/CCR7−) subpopulations (Additional file 1: Fig. S1C). Our data showed that the frequency of Naïve, TCM, TEM and TEMRA CD4+ T cell subpopulations did not change during the use of anti-TB therapy (Additional file 1: Fig. S2).

Together, these results suggest that the number of CD4+ T cells is increased during both DS- and DR-TB at 6 m of anti-TB therapy. However, CD4+ T cells from DR-TB decrease the expression of the TNF molecules pathway due to anti-TB therapy.

### In the 6th month of anti-TB therapy, DR-TB has increased TGFβ1 and IL-35 levels, but they also display a persistent pro-inflammatory cytokines profile

Our data showed that at the 6th month of anti-TB therapy, the frequency of CD4+ T cell subpopulation was recurrently affected in DS-TB and DR-TB; moreover, TNFR2 expression is decreased on the cell surface of uTreg, actCD4+ and CD4+ T cells, suggesting that TNFR2 pathway-dependent functions could be affected, for instance, regulatory functions of inflammation. Thus, we hypothesised that the inflammatory status between DS- and DR-TB is different at 6 m of anti-TB therapy use.

**Fig. 5:**
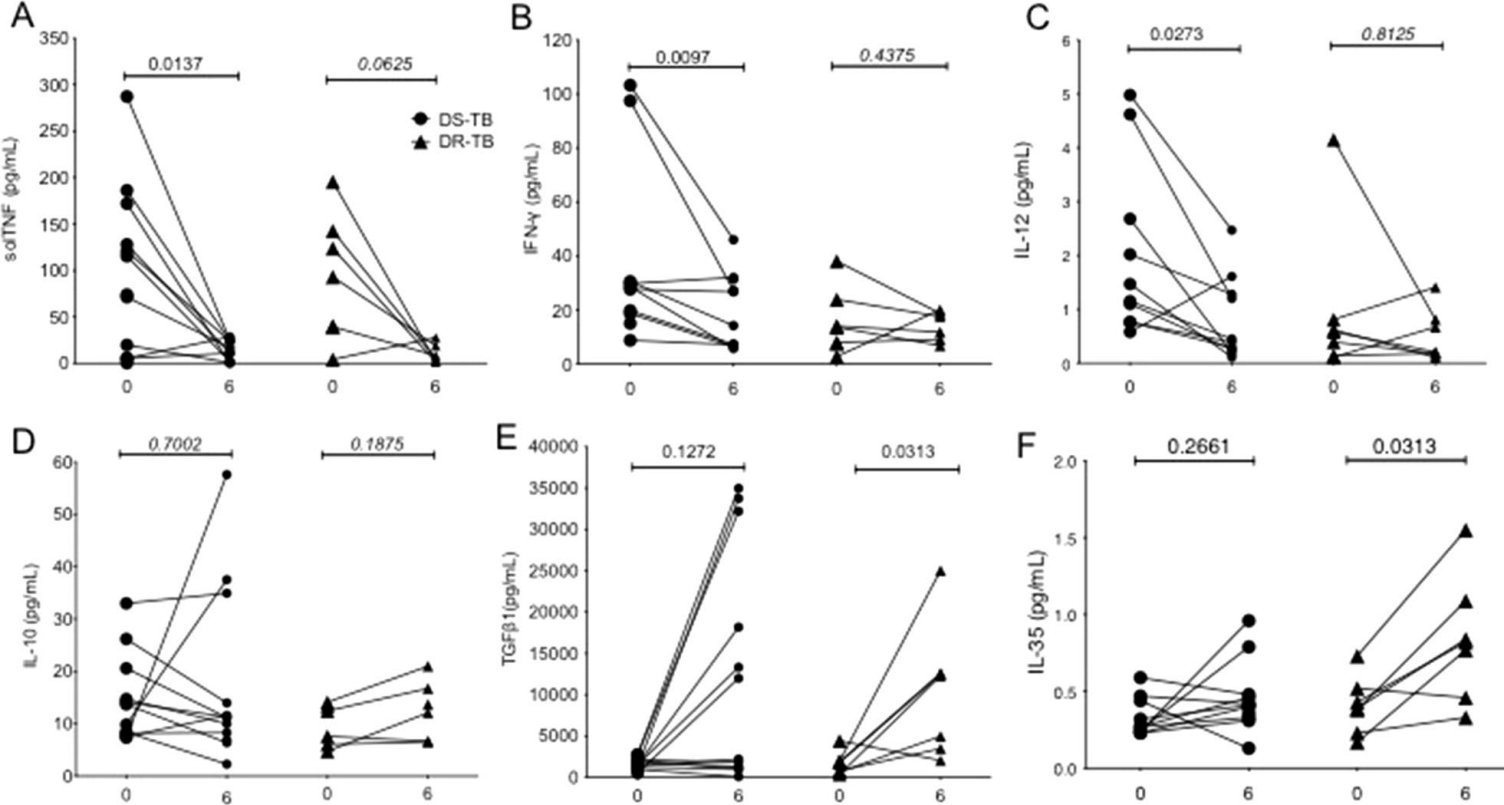
Pro-inflammatory cytokines keep increased in the sixth month of anti-TB therapy in DR-TB. Levels of cytokines from DS-TB and DR-TB patients were measured in plasma at diagnosis time (0) and 6 months (6) of starting the anti-TB therapy. Pro-inflammatory cytokines: sTNF (**A**), IFNγ (**B**), and IL-12 (IL12p70) (**C**), and anti-inflammatory cytokines: IL-10 (**D**), TGFβ1 (**E**), and IL-35 (**F**) were measured. DS-TB = 13, DR-TB = 7. Dots plots before-after. Wilcoxon matched-pairs test (*p < 0.05)

To confirm our hypothesis, the level of pro-inflammatory cytokines such as solTNF, IFNγ and IL-12 were evaluated in plasma from DS- and DR-TB patients at the basal time and 6 m anti-TB therapy (Fig. 5A–C, respectively). Results showed that DR-TB patients still display a high plasma level of IFNγ and IL-12 at 6 m of anti-TB therapy, whereas DS-TB patients showed a decrease in these cytokine levels (Fig. 5B and C, respectively). DR-TB patients also had a decreased solTNF level; however, it was not statistically significant (p = 0.0625). On the contrary, DS-TB patients significantly decreased solTNF level due to anti-TB therapy (p = 0.0137) (Fig. 5A). It suggests that even if both DS-TB and DR-TB showed decreased Treg frequency and tmTNFR2 expression is altered, DS-TB patients regulate the inflammatory status at 6 months of anti-TB therapy, whereas DR-TB patients are unable to regulate the inflammation.

Previous reports indicated high plasma levels of IL-10 in active TB patients because Treg is the primary source of IL-10 and TGFβ1 (Pinheiro et al. 2013; Josefowicz et al. 2012; Hadaschik and Enk 2015). IL-35 is a heterodimer composed of p35 (IL-12A) and Ebi3 (Epstein-Barr virus-induced gene 3); Treg is the primary source of IL-35, and it has solid suppressive properties both in vivo and in vitro. Recently, it has been demonstrated that IL-35- and IL-10-producing Treg have a different activation status and work in a complementary way to maintain immune tolerance (Collison et al. 2007; Wei et al. 2017). Considering our results regarding the alteration in the frequency of cTreg and uTreg, we evaluated the plasma level of IL-10, TGFβ1 and IL-35 (Fig. 5D–F, respectively).

The data showed that IL-10 plasma level is not modified in DS-TB and DR-TB to respond to anti-TB therapy (Fig. 5D). Interestingly, TGFβ1 and IL-35 plasma levels increased in DR-TB at 6 m of anti-TB therapy compared to basal time (Fig. 5E and F, respectively). This data suggests that DR-TB displays an increased cytokine level related to Treg due to anti-TB therapy despite having a high level of pro-inflammatory cytokines. DS-TB patients did not increase the cytokines' level related to Treg, probably because they also showed decreased Treg frequency.

### In the 6th month of anti-TB therapy, DR-TB has up-regulated TNF and TNFR2, whereas DS- and DR-TB down-regulate TNFR1, TGFβ1 and FOXP3 at the transcriptional level

**Fig. 6:**
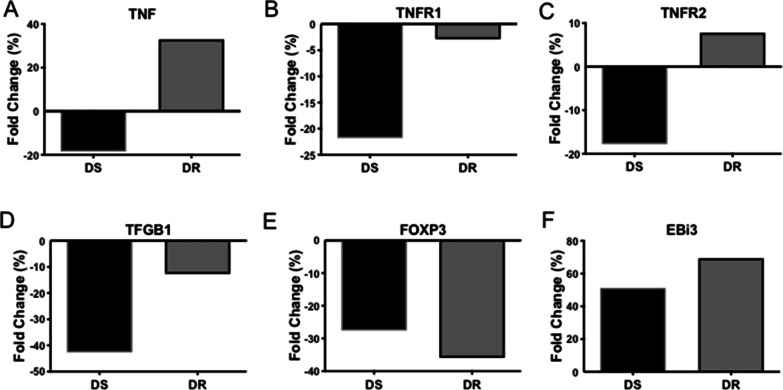
CD4+ T cells of DR-TB have decreased TNFR1, TGFβ1, and FOXP3 gene expression, even though TNF, TNFR2, and EBi3 expression is up-regulated at the transcriptional level. We calculated the fold change percentage of expression of TNF (**A**), TNFR1 (**B**), TNFR2 (**C**), TGFβ1 (**D**), FOXP3 (**E**), and EBi3 (**F**). The percentage of fold change was calculated by obtaining the difference between six months and before of anti-TB therapy per each group. DS-TB n = 3, DR-TB n = 3. Data are shown in bar graphs with median values

We evaluated TNF, TNFR1, TNFR2, FOXP3, TGFβ1 and EBi3 expression at the transcriptional level (Fig. 6), and their relative gene expression was evaluated in CD4+ lymphocytes from both DS-TB and DR-TB patients.

Taking the transcriptional level of basal time as a reference, our data shows that at the 6th month of anti-TB therapy, DS-TB patients present a negative fold change in TNF, TNFR1, TNFR2 molecules (Fig. 6A–C, respectively). In contrast, DR-TB patients present a positive fold change in TNF and TNFR2 transcriptional level in response to anti-TB therapy (Fig. 6A and C, respectively). Although DR-TB patients also showed a negative fold change to TNFR1, this drop was near 80% less dramatic than the negative fold change observed in DS-TB (Fig. 6B). This data suggests that DS-TB, at the transcriptional level, regulates TNF pathway molecules negatively due to anti-TB therapy; however, DR-TB patients cannot normalise the transcriptional level expression of TNF pathway molecules, keeping the high expression of these inflammatory molecules.

Regarding the regulation of molecules related to Treg cells' generation, TGFβ1, FOXP3, and EBi3 were evaluated at the transcriptional level at 6 months of anti-TB therapy (taking as a reference the transcriptional level at the basal time) (Fig. 6D–F, respectively). We observed that both DS- and DR-TB patients induced a similar profile in regulating these molecules; TGFβ1 and FOXP3 showed a negative fold change (Fig. 6D, E), whereas EBi3 is up-regulated (Fig. 6F).

## Discussion

Although the discovery of the causing agent of TB by Robert Koch was reported more than a century ago, the current TB field still needs the implementation of more effective therapies and vaccines than the current ones, as well as the identification of biomolecules that are helpful for earlier diagnostics and to better follow-up patients.

In this way, the study of TB immunopathogenesis is central to develop new tools for TB control worldwide. At present, it has been proposed that the immune response is different in subjects with DR-TB compared to DS-TB. However, there is not enough information describing molecules related to immune-regulation mechanisms to differentiate between infection with drug-susceptible and -resistant Mtb-strains and how the anti-TB treatment modulates those molecules.

This study showed that 6 months of anti-TB therapy decreases the frequency of cTreg tmTNF+, CD4+ tmTNFR1+, and CD4+ tmTNFR2+ cells in both DS- and DR-TB patients. However, only DR-TB decreases the frequency of actCD4+ tmTNF+ and actCD4+ tmTNFR2+ cells and exhibits a systemic inflammatory status characterised by high levels TNF, IFN-γ, and IL-12. This inflammatory status is most likely a consequence of the up-regulation at the transcriptional level of TNF and TNFR2, plus the negative regulation of TGFβ1 and FOXP3 shown in total CD4+ T cells from these patients.

The role of Treg has been deeply studied in several diseases; in vitro studies and some drugs have been evaluated that could be successful as adjuvant therapy to modulate the response of effector Th1 and Tregs (Tonby et al. 2016). During drug-resistant Mtb infection, it is not clear if the anti-TB therapy modifies the quality and quantity of total Treg, cTreg or uTreg; apparently, the presence of Treg during TB is necessary to improve a balanced immune response (Ahmed and Vyakarnam 2020).

A murine model has suggested that Treg plays a beneficial role by avoiding the tissues damage induced by excessive inflammation, but Treg prevents Mtb eradication by suppressing other CD4+ T cell response (Kursar et al. 2007). These reports demonstrate the interplay's relevance between CD4+ T cell subpopulations to preserve a balanced immune response. In this context, we demonstrated a decrease in the frequency of cTreg cells at 6 months of anti-TB therapy, and in contrast, the frequency of CD4+ T cells increased. This result suggests that anti-TB therapy tries to induce a balance of CD4+ T cell subpopulations, apparently through quantity, perhaps as a first effort to avoid an excessive inflammatory status, which is not beneficial for the host.

A previous report indicated that the frequency of Treg decreases after treatment in active DS-TB and Multi-Drug-resistant tuberculosis (MDR-TB) patients (Lim et al. 2013). Our data showed that although DR- and DS-TB patients have a decreased frequency of cTreg and cTreg tmTNF+ cells due to anti-TB therapy, only DS-TB patients have a decreased frequency of uTreg and uTregtmTNFR2+ cells. Previous reports in a murine model of mycobacterial infection showed that tmTNF expression is necessary on myeloid-derived suppressor cells' cell surface to induce a regulatory function (Chavez-Galan et al. 2017). We did not evaluate the suppressor function of cTreg or uTreg cells, but probably, the imbalance in the expression of the TNF pathway could modulate this function, and consequently, the regulation of the inflammation is affected.

We reported for the first time that, in response to 6 m of anti-TB therapy, only DR-TB, and not DS-TB patients, depicts a decrease in the frequency of actCD4+ tmTNF+ and actCD4+ tmTNFR2+ cells. Probably, it helps to induce a systemic inflammatory status for a long time (after 6 months of therapy). Together, data supports our hypothesis that the TNF pathway molecules are also necessary to maintain a proper suppressor function. To this end, we measured the relative expression of TNF molecules, and interestingly the fold change percentage showed that CD4+ T cells from DR-TB have a downregulation of the proteins TNFR1, FOXP3, and TGFβ1 when comparing the expression level before and after 6 months of anti-TB therapy. Moreover, TNF, TNFR2, and EBi3 are up-regulated only in DR-TB patients after 6 months of anti-TB therapy, suggesting that DR-TB treatment is slower than DS-TB therapy to regulate the expression of molecules necessary to modulate the inflammatory process.

It is essential to note that both DR-TB and DS-TB patients had a negative sputum culture since the second month of anti-TB therapy and had good adherence to treatment. Therefore, adequate follow-up in these patients is relevant because if DR-TB patients suspend anti-TB therapy at the 6th month, they have high possibilities of relapse, and it is one reason why the international guidelines recommend between 18 to 20 months of anti-TB therapy (WHO 2019). In this context, our results provide evidence to support that there is a slow host immune response during the therapy in DR-TB patients, and it is favouring a systemic inflammatory status displayed. This disturbed immune response is in agreement with the tissue damage observed in these patients and has been associated with the disease's chronicity.

Studies in murine models have shown that TNF neutralisation and anti-TB therapy favour decreasing the bacillary load and damage lung parenchyma (Bourigault et al. 2013). Nevertheless, we observed this decline only in DS-TB patients, whereas DR-TB patients had high levels of both sTNF and IFNγ even at the 6th month. This result is in concordance with a recent study where it is reported that DR-TB patients still have a high plasma level of IFN-γ and TNF for a longer time than DS-TB patients (Ocaña-Guzman et al. 2021). Therefore, we considered that the alteration in the immune response regulation in DR-TB patients could be explained, at least partially, by high production of other anti-inflammatory cytokines, such as TGFβ1 and IL-35, considering these cytokines remain increased in DR-TB patients at the 6th month as well.

The increased relative expression of the proteins TNF and TNFR2 in DR-TB patients could explain the chronic phase in these subjects; even though sputum culture was negative to Mtb, there is a constant alteration in the immune system, and if the treatment is stopped, there is a higher risk for disease relapse and development of XDR-TB.

Our results indicate that, as a response to anti-TB therapy, DR-TB decreases the frequency of cTreg cells and increases the frequency of CD4+ T cells. More interestingly, DR-TB patients decrease the frequency of actCD4+ tmTNF+ and actCD4+ tmTNFR2+ cells. Together, these findings suggest that DR-TB patients maintain a strong disturbance of the immune response, even with an appropriate treatment scheme (because they have negative sputum culture), enhancing the need to maintain the therapy during a long period.

On the contrary, a previous study reported that DR-TB patients had decreased expression of FOXP3, and authors suggested that when these patients received treatment together with rhIL-2, Th1 cells were increased, and Th17 and Treg populations were decreased, highlighting a clinical example for novel target immunotherapy (Tan 2017). Regarding this, the evaluation of other cytokines, such as IL-17, should be considered in future research, as high levels of IL-17 were observed in CD4+ T cells from MDR-TB patients infected with a resistant strain (Basile 2017). In studies with MDR Mtb isolates, such as the F15/LAM4/KZN strain, it has been demonstrated, in vitro*,* that MDR Mtb isolates induced the highest concentration of TNF in pulmonary epithelial cells in contrast with the Beijing strain; the authors suggested that these strains are less virulent because they do not subvert the host response (Mvubu et al. 2018). We propose that it is essential to consider these differences between strains, and more studies could be undertaken in vitro using cellular infection with drug-resistant strains to demonstrate alteration in TNF pathways in Treg cells. Another new field of research is the study of latent TB contacts with DR-TB patients and people with other comorbidities like diabetes mellitus, and these subjects should be included in more studies and to assess their immune response to controlling the infection.

One of the most important limitations in our study is the small sample size, but the subjects in our study received an integral evaluation every month by clinician experts. Therefore, we can confirm that both TB groups had a correct adherence to the treatment, and their AFB turned negative at a similar time. Moreover, although both TB groups had diabetes as the main comorbidity, we can confirm that it did not induce TB/diabetes patients to have a negative AFB at different times than TB/without diabetes patients. Furthermore, during the follow-up period, no patients developed clinical complications (e.g. kidney injury, diabetic ketoacidosis). Therefore, our results could be directly related to the origin of infection (DR vs DS Mtb strains) and anti-TB therapy, although more studies are needed to confirm our results in the TB field.

## Conclusion

In summary, evaluating the frequency of cTreg and uTreg cells could be a helpful tool for understanding the immune-regulatory status during DR-TB versus DS-TB therapy. Moreover, a complete profile to evaluate a better DR-TB status should include evaluating molecules related to Treg, both at the transcriptional and soluble protein level, because this profile favours a systemic inflammatory status. Our data indicate that the immune system regulation between DS-TB and DR-TB patients is different, and it may be considered during anti-TB therapy. Despite the 6th month point of treatment, DR-TB patients still show abnormalities in the immune system; they had a negative sputum culture. Thus, we propose that the measurement of other biomarkers in serum or plasma should be implemented for treated patients' follow-up. Also, new therapies to correct these immunological deregulations should be developed for DR-TB cases.

## Supplementary Information

**Additional file 1: Table S1.** Profiles of Drug-Resistant TB patients. **Fig. S1.** Representative flow cytometric analysis of PBMC in subjects with DS-TB and DR-TB.  Peripheral mononuclear cells (PBMC) were analyzed by flow cytometry, representative analysis of one subject is showed. Cells gate was selected on the base of forward scatter (FSC)/side scatter (SSC). Then, singles FSC ad SSC dot plot were realized, posteriorly live cells gate was restricted (Pe-Texas Red negative), and CD3+CD4+ lymphocytes cells were identified. From the CD3+CD4+ gate, the co-expression of CD25+ and FOXP3 was measured to identify four subpopulations; activated CD4+ T cells (Q1: CD4+CD25+FOXP3-), conventional Treg cells (Q2: CD4+CD25+FOXP3+), unconventional Treg cells (Q3: CD4+CD25-FOXP3+), and others CD4+ T cells (Q4: CD4+CD25-FOXP3-) (A). Then, the expressions of tmTNF, tmTNFR1, and tmTNFR2 were measured in each CD4+ subpopulation (black histogram), FMO was used to identify the background signal (gray histogram) (B). Finally, from live cells gate, CD3+CD4+ cells were identified, and then the expressions CD45RA and CCR7 expression were evaluated: Naïve (CD45RA+/CCR7+), central memory (TCM, CD45RA-/CCR7+), effector memory (TEM, CD45RA-/CCR7-), and effector memory RA (TEMRA, CD45RA+/CCR7-) (C). **Fig. S2. **TEMRA and Naïve CD4+ T cells subpopulations are lower in DR-TB than in DS-TB after anti-TB therapy. Peripheral mononuclear cells (PBMC) from DS-TBand DR-TB patients were obtained at diagnosis time (basal), 2 months (2m) and 6 months (6m) of starting the anti-TB therapy, PBMC were prepared for flow cytometry. Then, frequency of CD4+ cell populations were measured: TEMRA cells (CD4+CD45RA+CCR7-) (A), Naïve cells (CD4+CD45RA+CCR7+) (B), EM cells (CD4+CD45RA-CCR7+)(C), and CM cells (CD4+CD45RA-CCR7-) (D). DS-TB=11, DR-TB=7. Bar graphs showing means ± SEM. One Way ANOVA with Dunn's post-test multiple comparisons tests.

## Data Availability

The authors confirm the raw data to support this study's conclusions are included in the manuscript (as a primary text or supplemental material). The corresponding author will provide more information, upon rational request, to any qualified researcher.

## Abbreviations

AFB: Acid-fast bacilli smear test
cTreg: Conventional regulatory T cell
DS-TB: Drug-susceptible tuberculosis
DR-TB: Drug-resistant tuberculosis
Ebi3: Epstein-Barr virus-induced gene 3
Foxp3: Factor forkhead box P3
IL: Interleukin
M. bovis: *Mycobacterium bovis*
MDR-TB: Multi-drug-resistant tuberculosis
Mtb: *Mycobacterium tuberculosis*
PBMC: Peripheral blood mononuclear cell
TACE: TNF-α converting enzyme
TB: Tuberculosis
TGFβ: Transforming growth factor beta
TNF: Tumor necrosis factor
TNFR1: Tumor necrosis factor receptor 1
TNFR2: Tumor necrosis factor receptor 2
Treg: Regulatory T cell
uTreg: Unconventional regulatory T cell
XDR-TB: Extensively drug-resistant tuberculosis
