# Supplementary material for: Anti-tuberculosis chemotherapy alters TNFR2 expression on CD4+ lymphocytes in both drug-sensitive and -resistant tuberculosis: however, only drug-resistant tuberculosis maintains a pro-inflammatory profile after a long time
Source: Mol Med. 2021 Jul 14;27:76. doi: 10.1186/s10020-021-00320-4 (PMC8278684; doi:10.1186/s10020-021-00320-4)
Supplement: Supplementary file 1 — Additional file 1: Table S1. Profiles of Drug-Resistant TB patients. Fig. S1. Representative flow cytometric analysis of PBMC in subjects with DS-TB and DR-TB. Peripheral mononuclear cells (PBMC) were analyzed by flow cytometry, representative analysis of one subject is showed. Cells gate was selected on the base of forward scatter (FSC)/side scatter (SSC). Then, singles FSC ad SSC dot plot were realized, posteriorly live cells gate was restricted (Pe-Texas Red negative), and CD3+CD4+ lymphocytes cells were identified. From the CD3+CD4+ gate, the co-expression of CD25+ and FOXP3 was measured to identify four subpopulations; activated CD4+ T cells (Q1: CD4+CD25+FOXP3-), conventional Treg cells (Q2: CD4+CD25+FOXP3+), unconventional Treg cells (Q3: CD4+CD25-FOXP3+), and others CD4+ T cells (Q4: CD4+CD25-FOXP3-) (A). Then, the expressions of tmTNF, tmTNFR1, and tmTNFR2 were measured in each CD4+ subpopulation (black histogram), FMO was used to identify the background signal (gray histogram) (B). Finally, from live cells gate, CD3+CD4+ cells were identified, and then the expressions CD45RA and CCR7 expression were evaluated: Naïve (CD45RA+/CCR7+), central memory (TCM, CD45RA-/CCR7+), effector memory (TEM, CD45RA-/CCR7-), and effector memory RA (TEMRA, CD45RA+/CCR7-) (C). Fig. S2. TEMRA and Naïve CD4+ T cells subpopulations are lower in DR-TB than in DS-TB after anti-TB therapy. Peripheral mononuclear cells (PBMC) from DS-TBand DR-TB patients were obtained at diagnosis time (basal), 2 months (2m) and 6 months (6m) of starting the anti-TB therapy, PBMC were prepared for flow cytometry. Then, frequency of CD4+ cell populations were measured: TEMRA cells (CD4+CD45RA+CCR7-) (A), Naïve cells (CD4+CD45RA+CCR7+) (B), EM cells (CD4+CD45RA-CCR7+)(C), and CM cells (CD4+CD45RA-CCR7-) (D). DS-TB=11, DR-TB=7. Bar graphs showing means ± SEM. One Way ANOVA with Dunn's post-test multiple comparisons tests. [file 10020_2021_320_MOESM1_ESM.docx]

**Title:** Anti-tuberculosis chemotherapy alters TNFR2 expression on CD4+ lymphocytes in both drug-sensitive and -resistant tuberculosis. However, only drug-resistant tuberculosis maintains a pro-inflammatory profile after a long time**.**

**Running title**: Anti-TB therapy decreases TNFR2 expression and favours pro-inflammatory cytokines in DR-TB.

**Authors:**

Norma A. Tellez-Navarrete^1^, Lucero A. Ramon-Luing^1^, Marcela Muñoz-Torrico^2^, Mario García-Preciado^1^, Karen Medina-Quero^3^, Rogelio Hernandez-Pando^4^, Leslie Chavez-Galan^1*^

**Adscription**

^1^ Laboratory of Integrative Immunology, Instituto Nacional de Enfermedades Respiratorias “Ismael Cosío Villegas”, Mexico City, Mexico.

^2^ Clinic of Tuberculosis, Instituto Nacional de Enfermedades Respiratorias “Ismael Cosío Villegas”, Mexico City, Mexico

^3^ Laboratory of Immunology, Escuela Militar de Graduados en Sanidad, Mexico City, Mexico.

^4^ Experimental Pathology Section, Department of Pathology, Instituto Nacional de Ciencias Médicas y Nutrición Salvador Zubirán, Mexico City, Mexico.

***Corresponding Author**:

Leslie Chávez-Galán, PhD.

Adress: Laboratorio de Inmunología Integrativa, Instituto Nacional de Enfermedades Respiratorias Ismael Cosío Villegas. Calzada de Tlalpan No. 4510.Mexico City, Mexico. CP. 14080

Phone.: +52 55 54871700 Ext. 5270 Fax: +52 55 56654623

E-mail: [lchavez_galan@iner.gob.mx](mailto:lchavez_galan@iner.gob.mx)

**Supplementary material**

**Supplementary Table 1. Profiles of Drug-Resistant TB patients.**

| **Resistant Drug** | **N=7(%)** |
| --- | --- |
| Rifampicin | 4 (58%) |
| Rifampicin + Pirazinamide | 1(14%) |
| Rifampicin + Isoniazid | 1 (14%) |
| Rifampicin + Isoniazid + Moxifloxacin | 1 (14%) |


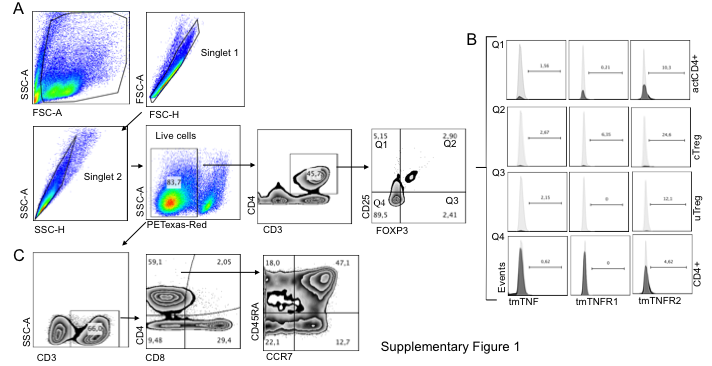


**Supplementary Figure S1. Representative flow cytometric analysis of PBMC in subjects with DS-TB and DR-TB.**  Peripheral mononuclear cells (PBMC) were analyzed by flow cytometry, representative analysis of one subject is showed. Cells gate was selected on the base of forward scatter (FSC)/side scatter (SSC). Then, singles FSC ad SSC dot plot were realized, posteriorly live cells gate was restricted (Pe-Texas Red negative), and CD3+CD4+ lymphocytes cells were identified. From the CD3+CD4+ gate, the co-expression of CD25+ and FOXP3 was measured to identify four subpopulations; activated CD4+ T cells (Q1: CD4^+^CD25^+^FOXP3^-^), conventional Treg cells (Q2: CD4^+^CD25^+^FOXP3^+^), unconventional Treg cells (Q3: CD4^+^CD25^-^FOXP3^+^), and others CD4+ T cells (Q4: CD4^+^CD25^-^FOXP3^-^) (A). Then, the expressions of tmTNF, tmTNFR1, and tmTNFR2 were measured in each CD4+ subpopulation (black histogram), FMO was used to identify the background signal (gray histogram) (B). Finally, from live cells gate, CD3+CD4+ cells were identified, and then the expressions CD45RA and CCR7 expression were evaluated: Naïve (CD45RA+/CCR7+), central memory (TCM, CD45RA-/CCR7+), effector memory (TEM, CD45RA-/CCR7-), and effector memory RA (TEMRA, CD45RA+/CCR7-) (C).


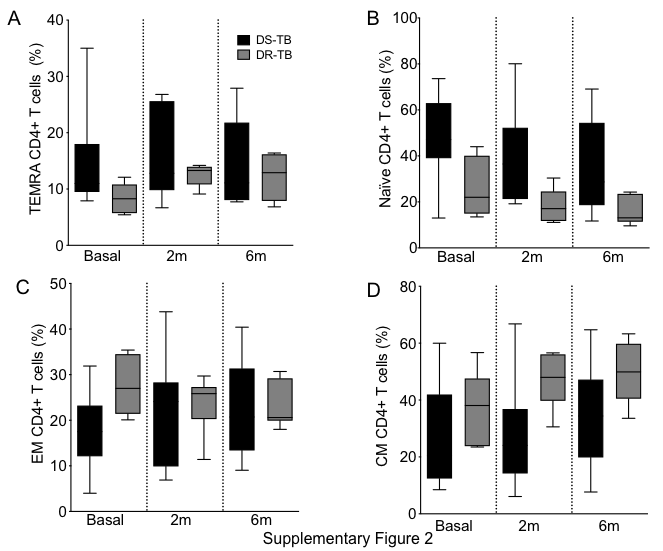


**Supplementary Figure S2. TEMRA and Naïve CD4+ T cells subpopulations are lower in DR-TB than in DS-TB after anti-TB therapy.** Peripheral mononuclear cells (PBMC) from DS-TB and DR-TB patients were obtained at diagnosis time (basal), two months (2m) and six months (6m) of starting the anti-TB therapy, PBMC were prepared for flow cytometry. Then, frequency of CD4+ cell populations were measured**:** TEMRA cells (CD4+CD45RA+CCR7-) (A), Naïve cells (CD4+CD45RA+CCR7+) (B), EM cells (CD4+CD45RA-CCR7+) (C), and CM cells (CD4+CD45RA-CCR7-) (D). DS-TB=11, DR-TB=7. Bar graphs showing means ± SEM. One Way ANOVA with Dunn's post-test multiple comparisons tests.
